# NOX2 oxidase expressed in endosomes promotes cell proliferation and prostate tumour development

**DOI:** 10.18632/oncotarget.26237

**Published:** 2018-10-23

**Authors:** Ian P. Harrison, Antony Vinh, Ian R.D. Johnson, Raymond Luong, Grant R. Drummond, Christopher G. Sobey, Tony Tiganis, Elizabeth D. Williams, John J. O’ Leary, Doug A. Brooks, Stavros Selemidis

**Affiliations:** ^1^ Infection and Immunity Program, Biomedicine Discovery Institute, Department of Pharmacology, Monash University, Melbourne, Victoria 3800, Australia; ^2^ Department of Physiology, Anatomy and Microbiology, School of Life Sciences, La Trobe University, Melbourne, Victoria 3086, Australia; ^3^ School of Pharmacy and Medical Sciences, University of South Australia Cancer Research Institute, University of South Australia, Adelaide, South Australia 5001, Australia; ^4^ Metabolic Disease and Obesity Program, Biomedicine Discovery Institute, Department of Biochemistry and Molecular Biology, Monash University, Melbourne, Victoria 3800, Australia; ^5^ Australian Prostate Cancer Research Centre-Queensland, School of Biomedical Sciences, Institute of Health and Biomedical Innovation, Queensland University of Technology, Translational Resea rch Institute, Brisbane, Queensland 4000, Australia; ^6^ Histopathology, School of Medicine Trinity College Dublin, Ireland, Sir Patrick Dun’s Laboratory, Central Pathology Laboratory, St James’s Hospital, Dublin 8, Ireland; ^7^ Emer Casey Research Laboratory, Molecular Pathology Laboratory, The Coombe Women and Infants University Hospital, Dublin 8, Ireland; ^8^ Program in Chronic Infectious and Inflammatory Diseases, School of Health and Biomedical Sciences, College of Science, Engineering and Health, RMIT University, Bundoora, Victoria 3083, Australia

**Keywords:** NOX2, NADPH oxidase, endosome, reactive oxygen species, prostate cancer

## Abstract

Reactive oxygen species (ROS) promote growth factor signalling including for VEGF-A and have potent angiogenic and tumourigenic properties. However, the precise enzymatic source of ROS generation, the subcellular localization of ROS production and cellular targets *in vivo* that influence tumour-promoting processes, are largely undefined. Here, using mRNA microarrays, we show increased gene expression for NOX2, the catalytic subunit of the ROS-generating NADPH oxidase enzyme, in human primary prostate cancer compared to non-malignant tissue. In addition, NOX4 gene expression was markedly elevated in human metastatic prostate cancers, but not in primary prostate tumours. Using a syngeneic, orthotopic mouse model of prostate cancer the genetic deletion of NOX2 (i.e. NOX2^**-/y**^ mouse) resulted in reduced angiogenesis and an almost complete failure in tumour development. Furthermore, pharmacological inhibition of NOX2 oxidase suppressed established prostate tumours in mice. In isolated endothelial cells, and in human normal and prostate cancer cells, NOX2 co-located to varying degrees with early endosome markers including EEA1, Appl1 and Rab5A and the late endosome marker Rab7A, and this correlated with significant VEGF-A-dependent ROS production within acidified endosomal compartments and endothelial cell proliferation that was NOX2 oxidase- and hydrogen peroxide dependent. We concluded that NOX2 oxidase expression and endosomal ROS production were important for prostate cancer growth and that this was required to positively regulate the VEGF pathway. The research provides a paradigm for limiting tumour growth through a better understanding of NOX2 oxidase's effect on VEGF signalling and how controlling the development of tumour vasculature can limit prostate tumour development and metastasis.

## INTRODUCTION

A new paradigm in cancer cell research is that vascular endothelial growth factor VEGF signalling is dependent on receptor internalisation into early endosomes. Delays in the trafficking of VEGFR2-containing endosomes leads to VEGFR2 dephosphorylation, resulting in receptor de-activation and decreased arterial morphogenesis [18]. In both vascular and non-vascular cells, VEGFR2 undergoes constitutive recycling between peripheral endosomes and the cell surface [16]. VEGF in turn stimulates the production of reactive oxygen species (ROS) such as superoxide and hydrogen peroxide (H_2_O_2_) that promote cell proliferation and angiogenesis [32, 33]. H_2_O_2_ also stimulates VEGF production in vascular smooth muscle cells [26], and upregulates VEGF mRNA to induce endothelial cell proliferation and migration [34]. ROS are produced in a number of cell types, including tumour and endothelial cells [14], not only as by-products of normal cellular metabolism, but also as designated enzymatic products [17]. The NADPH oxidase (NOX) enzymes are a family of oxidases whose sole function is to generate ROS [2, 8, 27], and this appears to have an important role in angiogenesis [32, 28]. However, the exact enzymatic source of ROS generation and the subcellular location of ROS production remain largely unknown.

ROS are highly reactive molecules and therefore their site of generation strictly influences their site of action and consequences. Importantly, through spatial restriction, ROS avoid off target effects. There is emerging evidence that ROS production occurs in specific compartments of the cell in response to invading microorganisms. For example, it has been known for a long time that ROS production occurs in phagosomes in immune cells like macrophages and neutrophils [9]. This confinement allows for direct ROS action within this compartment that is critical for bacterial and fungal killing. More recently, key components of NADPH oxidase enzymes have been shown in endosomal membranes including the NOX1 and NOX2 catalytic subunits, as well as ROS-metabolising enzymes, like superoxide dismutase 3 (SOD3) [23, 30]. Moreover, the topology of the NOX-containing NADPH oxidase in the endosomal membrane is such that electrons are transferred from the cytosolic donor NADPH through the NOX catalytic subunit to produce superoxide in the endosomal lumen. Indeed, endosomal superoxide generation occurs via NOX2- and NOX1-containing NADPH oxidases [5, 23, 30] in response to varying stimuli including viruses that enter cells by endocytosis [30] and by extracellular stimuli such as receptor ligand complexes GPCRs and cytokines [20, 21]. Importantly, superoxide is rapidly converted to H_2_O_2_ by SOD within endosomal compartments and although the exact identities of the ROS responsible for redox signalling remain largely unexplored, it is presumed that H_2_O_2_ plays a major role. Furthermore, the role of endothelial endosomal NADPH oxidases in the context of cell proliferation and angiogenesis are yet to be defined.

Here we have investigated NOX2 and NOX4 gene expression in prostate cancer microarray databases and evaluated their gene expression in fresh-frozen tissue sections from radical prostatectomies. We also utilised a syngeneic mouse model of prostate cancer that allowed us to examine the role of host NOX2 expressed in the tumour microenvironment or stromal endothelial cells. Given that NADPH oxidases and VEGFR2 reside in endosomes, we hypothesized that VEGF causes ROS production within endosomes, and that this is an essential signalling platform for promoting cell proliferation and thereby angiogenesis in cancer. We concluded that NOX2 is integrally involved in prostate cancer development and should therefore be considered as a target for therapeutic intervention.

## RESULTS

### NOX2 and NOX4 expression profiles in human prostate cancers

**Figure 1:**
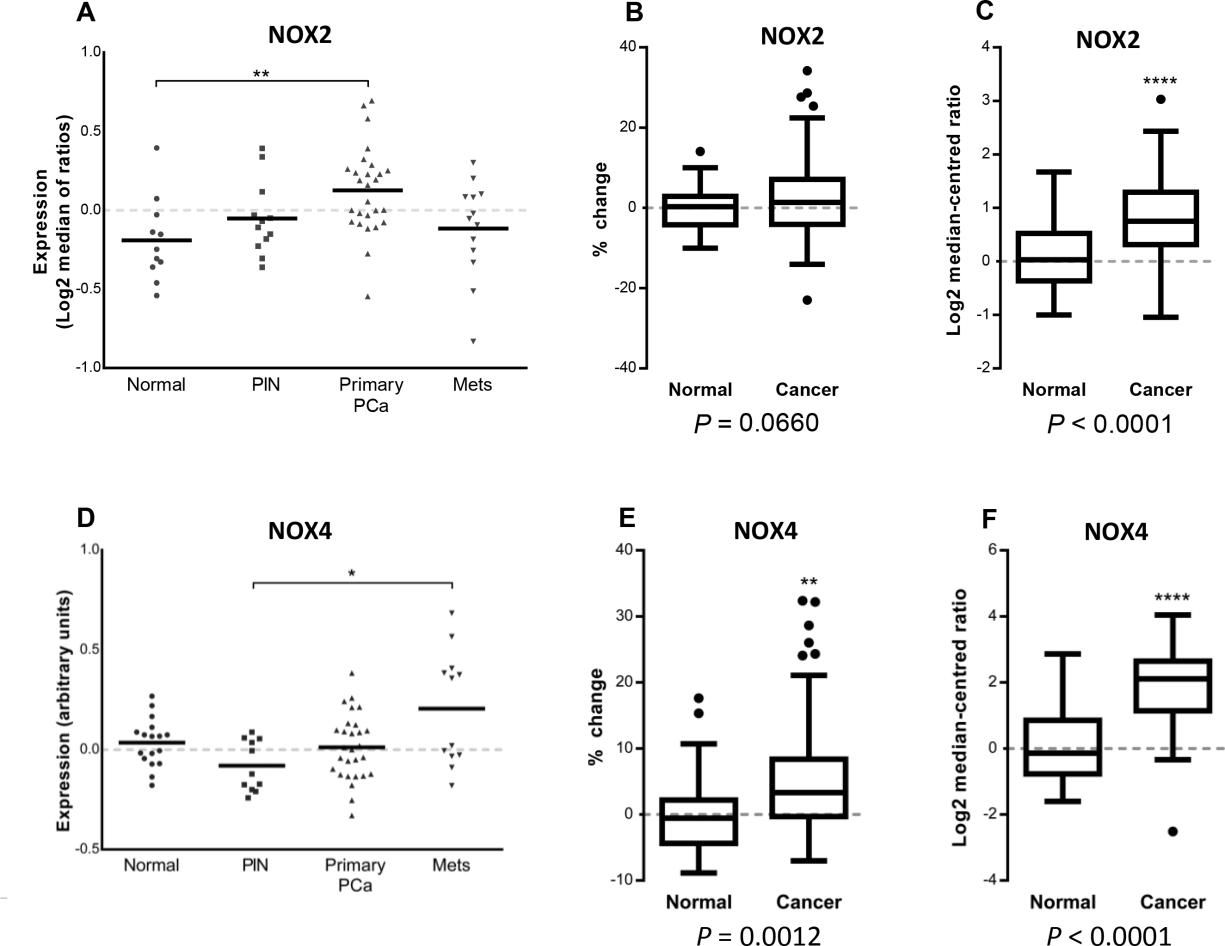
NOX2 and NOX4 expression in microarray database analyses of normal, primary and metastatic human prostate cancers Vertical scatter plot of NOX2 and NOX4 expression data from the Tomlins cohort (**A** and **D** respectively) consisting of 18 non-malignant tissues, 13 prostatic intraepithelial neoplasia’s, 30 primary prostate cancer and 19 metastatic cancer tissue samples. NOX2 and NOX4 expression displayed as percentage-change from the Taylor cohort (**B** and **E** respectively) consisting 29 non-malignant and 131 primary-cancer tissue samples. Log2 median-centred ratio of NOX2 and NOX4 from the Grasso cohort (**C** and **F** respectively) consisting 59 cancer 28 normal tissue samples. ^*^P < 0.05, ^**^P < 0.01 and ^****^P < 0.0001 for Students *t* test (C, E and F) or one-way ANOVA (A and D).

The expression of NOX2, when analysed from the Tomlins cohort, was significantly increased in primary prostate cancer tissue when compared with normal tissue (*P <* 0.01; Figure 1A). While overall, the expression of NOX2 in the Taylor cohort was not significantly increased in cancer tissue compared to non-malignant tissue cohort (*P* = 0.06; Figure 1B), NOX2 expression was significantly elevated in cancer tissue from the Grasso cohort (*p <* 0.0001; Figure 1C). The expression of NOX4, analysed from the Tomlins cohort, was significantly increased in metastatic tissue when compared with PIN (*P <* 0.05; Figure 1D). The cancer tissue from both the Taylor cohort (*P <* 0.01; Figure 1E) and the Grasso cohort (*P <* 0.0001; Figure 1F) displayed a significant increase in NOX4 expression.

### NOX2 oxidase promotes prostate tumour growth *in vivo*

**Figure 2:**
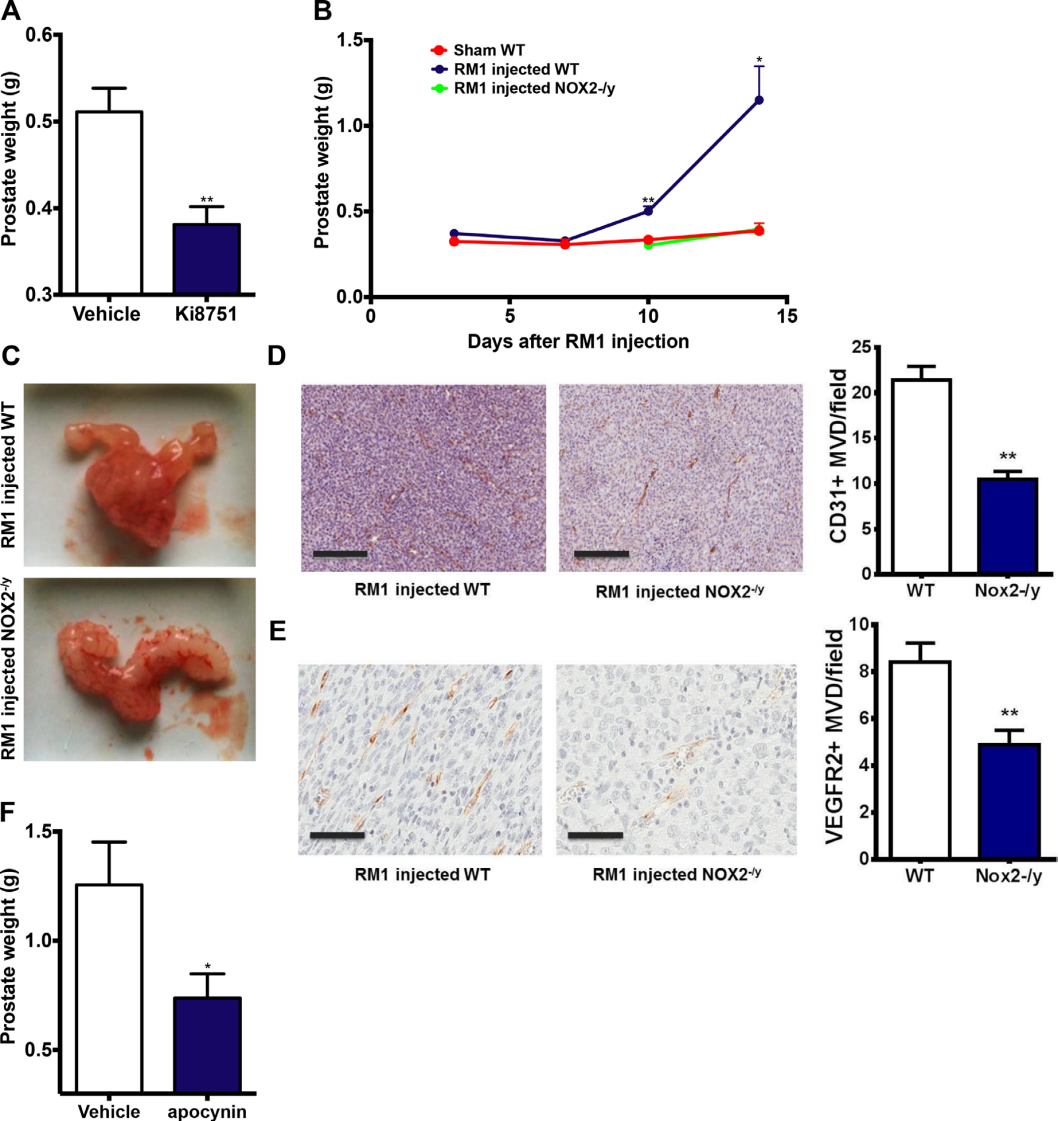
VEGFR2 and NOX2 activity are crucial for prostate tumour growth in mice (**A**) The effect of the VEGFR2 inhibitor Ki8751 (25 mg/kg/day, i.p) administered from Day 10 on prostate tumour growth in mice after 14 days (*n* = 8). (**B**) The data and (**C**) representative images showing the growth of prostate tumours over 14 days in WT and NOX2^-/y^ mice (*n* = 8–15). For (A) and (B) the prostate weights include the prostate and any associated tumour plus seminal vesicles. (**D**) Representative images of the density of CD31^+^ cells in prostate tumours in WT mice at Day 14. Note, the image in the NOX2^-/y^ represents the CD31+ staining in one of the larger tumours formed in the NOX2^-/y^ mice group. Horizontal black bar represents the scale of 200mm. The graph in (D) shows the average number of CD31^+^ cells per field in each group. (**E**) Representative images of the density of VEGFR2^+^ cells in prostate tumours in WT mice at Day 14. Horizontal black bar represents the scale of 200 mm. The graph in (E) shows the average number of VEGFR2^+^ cells per field in each group. (**F**) Group data showing effect of apocynin (50 mg/kg/day i.p. and 500 mg/L drinking water) on tumour development at Day 14 when administered in WT mice bearing tumours at Day 10 (*n* = 8). Data are mean ± SEM. Students unpaired *t* test (A, D, F) or one-way ANOVA (B and G). ^*^P < 0.05, ^**^P < 0.01.

To determine the contribution of NOX2 to tumour growth, we utilised a syngeneic cancer model. RM1 prostate cancer cells (10^4^ cells) were orthotopically implanted into the prostates of wild-type (C57BL/6J) mice, and after 10 and 14 days a significant tumour developed in the prostate. Note that sham prostates weighed ∼ 0.34 g. Initiating the treatment of tumour bearing WT mice at day 10 with the VEGFR2 inhibitor (Ki8751; 20 mg/kg/day i.p) significantly (*P <* 0.01) suppressed tumour development, compared to vehicle treatment, indicating that VEGFR2 drives tumour development in this animal model (Figure 2A). We observed a significant reduction (*P <* 0.01) in, and in some cases a complete absence of, tumours in NOX2^-/y^ mice injected with RM1 prostate cancer cells (Figure 2B and 2C). There was a significant reduction in angiogenesis in the prostate tumours of NOX2^-/y^ mice (*P <* 0.01 compared to WT) as assessed by anti-CD31 staining of endothelial cells (Figure 2D). There was also a significant reduction in the density of VEGFR2 expression in tumours of NOX2^-/y^ mice (Figure 2E). We next undertook a pharmacological approach to suppress NOX2 oxidase *in vivo,* in tumour bearing WT mice. Mice that were exposed to the NOX2 oxidase inhibitor and H_2_O_2_ scavenger apocynin (50 mg/kg/day i.p and 500 mg/L drinking water) from Day 10 displayed significantly smaller (*P <* 0.05; equating to ∼58% reduction in tumour size) prostate tumours at Day 14 than the controls (Figure 2F).

### Early and late endosomes co-locate with NOX2 and VEGFR2

**Figure 3:**
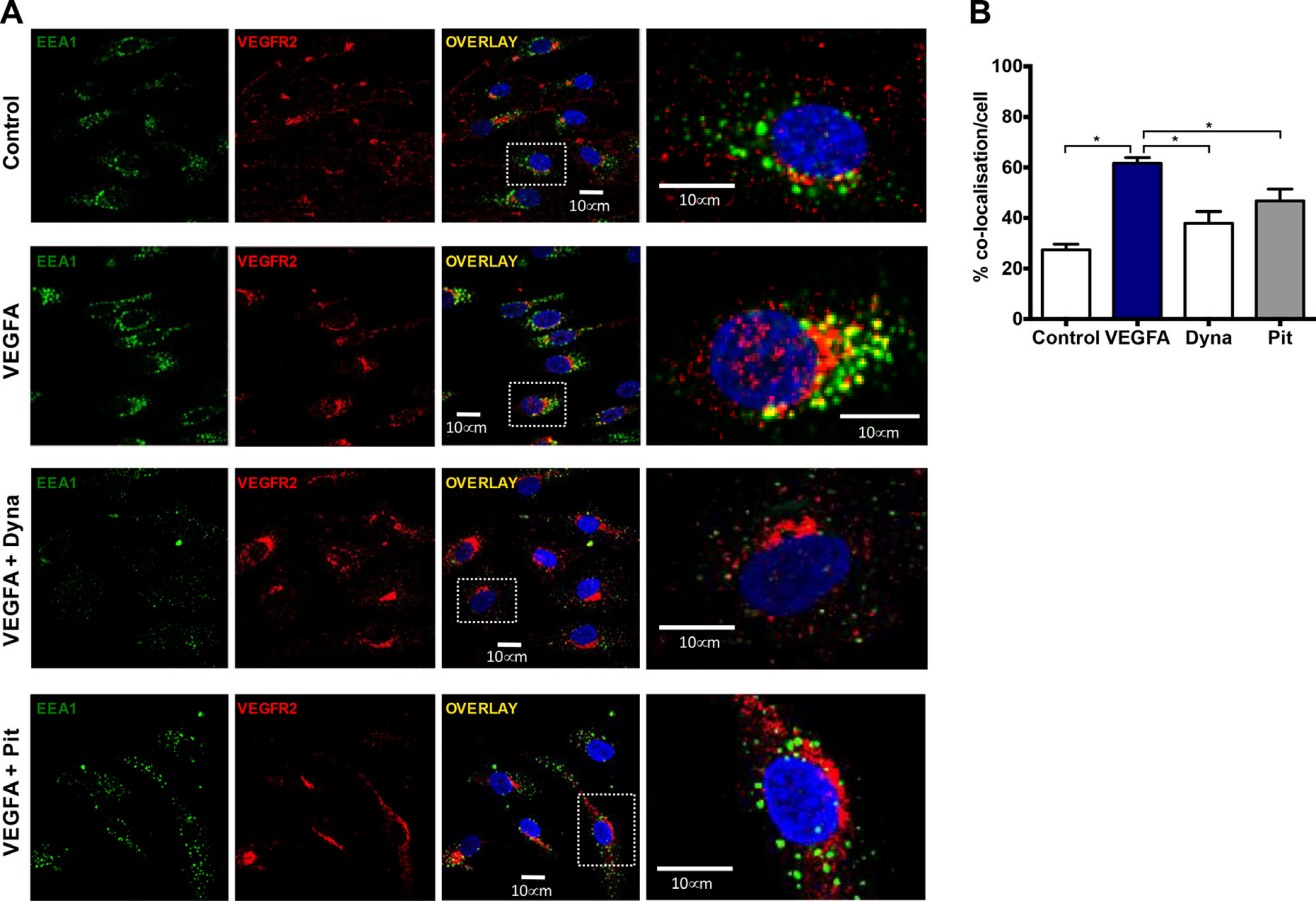
Co-location of early endosomes and VEGFR2 in the presence of VEGF is endocytosis-dependent (**A**) Confocal immunofluorescent images showing EEA1 and VEGFR2 co-location within HMEC-1 in and around the DAPI-stained nucleus after 30 min incubation with either PBS, VEGF-A (30 ng/mL), VEGF-A (30 ng/mL) plus dynasore (Dyna; 100 µM) or VEGF-A (30 ng/mL) plus Pitstop 2 (Pit; 30 µM). (**B**) Graphs show the degree of co-location within these cells in both groups expressed as percentage of total EEA1 positive staining. Data is representative of >50 cells imaged across 4 experiments and are shown as mean ± SEM. ^*^P < 0.05 for one-way ANOVA.

To investigate whether activated VEGFR2 internalizes into early endosomes, and if this leads to NOX2 oxidase activation, we treated human microvascular endothelial cells (HMEC-1) with either PBS (control) or VEGF-A (30 ng/mL) for 30 min and assessed co-location of VEGFR2 and EEA1. VEGF-A exposure resulted in increased co-location of VEGFR2 with EEA1 when compared with the controls (*P <* 0.05, *n* = 4; Figure 3A). Of note, there was still strong VEGFR2 staining that did not co-locate with EEA1, signifying co-locality in alternate subsets of endosomes. This VEGF-A stimulated co-location of EEA1 and VEGFR2 was abrogated by the endocytosis inhibitors, dynasore (100 µM; Figure 3B) and Pitstop 2 (30 µM; Figure 3). This indicates that VEGF-A exposure increases the co-location of VEGFR2 with intracellular endosomes placing this receptor in this important intracellular signalling compartment.

**Figure 4:**
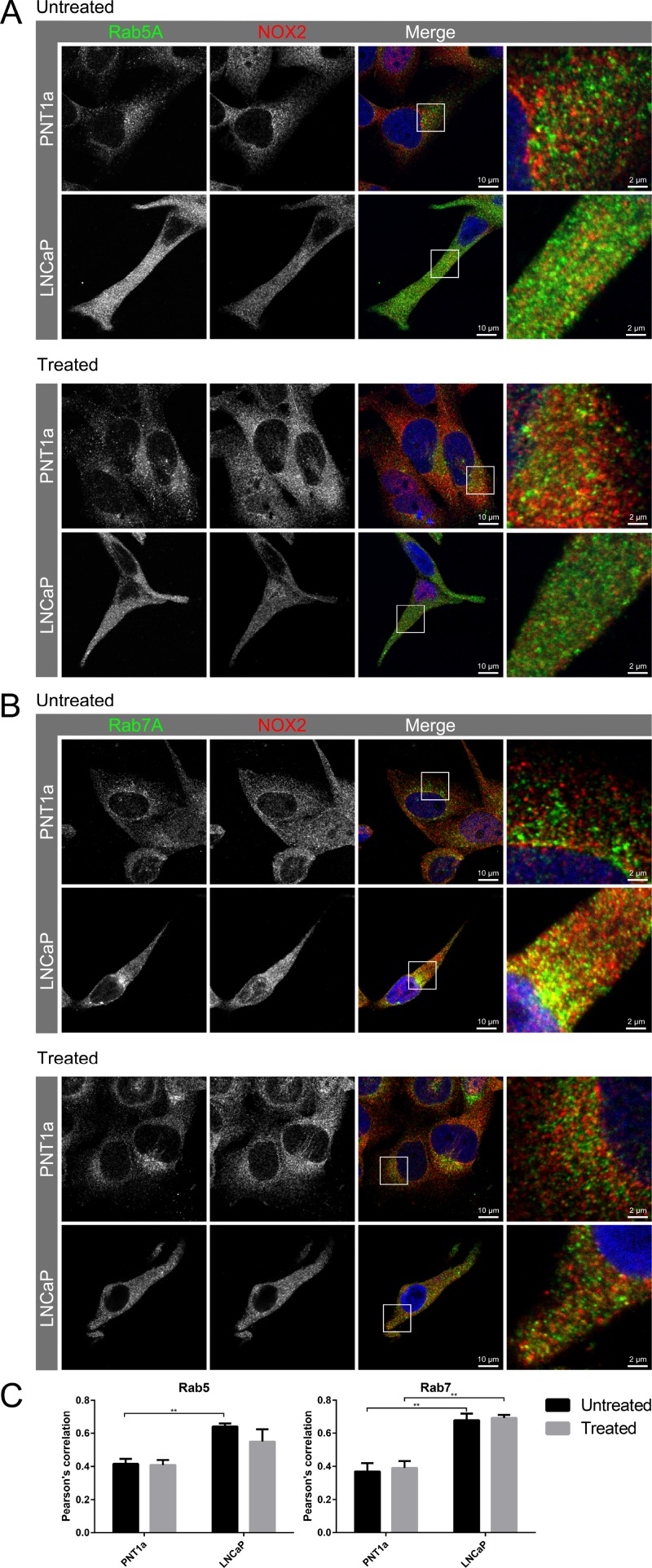
Co-location of NOX2 with endosome markers in response to VEGF-A treatment (**A**–**B**) Confocal fluorescent images showing co-located NOX2 (red) with endosome markers (green) (A) Rab5 and (B) Rab7 in non-malignant (PNT1A) and malignant (LNCaP) human prostate cancer cells and in LNCaP post-VEGF-A treatment. (**C**) Graphs show the degree of co-location of NOX2 with the endosome markers Rab5 and Rab7 in VEGF-A-treated and untreated PNT1a and LNCaP cells. Data is representative of 6 randomly selected cells and are shown as mean ± SEM. ^*^P < 0.05, ^**^P < 0.01 for two-way ANOVA.

We have recently shown that endosome biogenesis and function are altered in prostate cancer [15]. Indeed, Appl1-positive endosomes were detected throughout the cell cytoplasm of non-malignant control cells, whereas in prostate cancer cells these compartments were more concentrated at the cell periphery, particularly near the plasma membrane in cellular extensions/pseudopodia (Supplementary Figure 1). In addition, in non-malignant cells, both Rab5A and its effector EEA1 (Supplementary Figure 1) were concentrated in the perinuclear region, whereas in prostate cancer cells, these endosomal compartments were found throughout the cytoplasm, with some compartments located toward the cell periphery in cellular extensions (Figure 4). Rab7-positive endosomes were located mainly in the perinuclear region of both non-malignant and prostate cancer cells. We therefore examined the subcellular distribution of NOX2 in non-malignant and prostate cancer cells. NOX2 co-located with Appl1, Rab5A, EEA1 and Rab7 endosome markers in non-malignant cells, but the co-location of NOX2 with these different endosome compartments was significantly increased in prostate cancer cells (Figure 4 and Supplementary Figure 1). To investigate the effects of VEGF-A treatment on NOX2 co-location within endosomes, we treated cells with VEGF-A (30 ng/mL, 30 min) and assessed the co-location of NOX2 with Appl1, Rab5, EEA1 and Rab7 (Figure 4 and Supplementary Figure 1). There was no statistically significant change in the amount of endosome and NOX2 co-location in response to VEGF-A treatment (Figure 4). Thus, VEGF-A treatment did not increase the NOX2 distribution in endosomes, but there were more NOX2 endosome compartments in prostate cancer compared to non-malignant cells, signifying the potential to generate more ROS.

**Figure 5:**
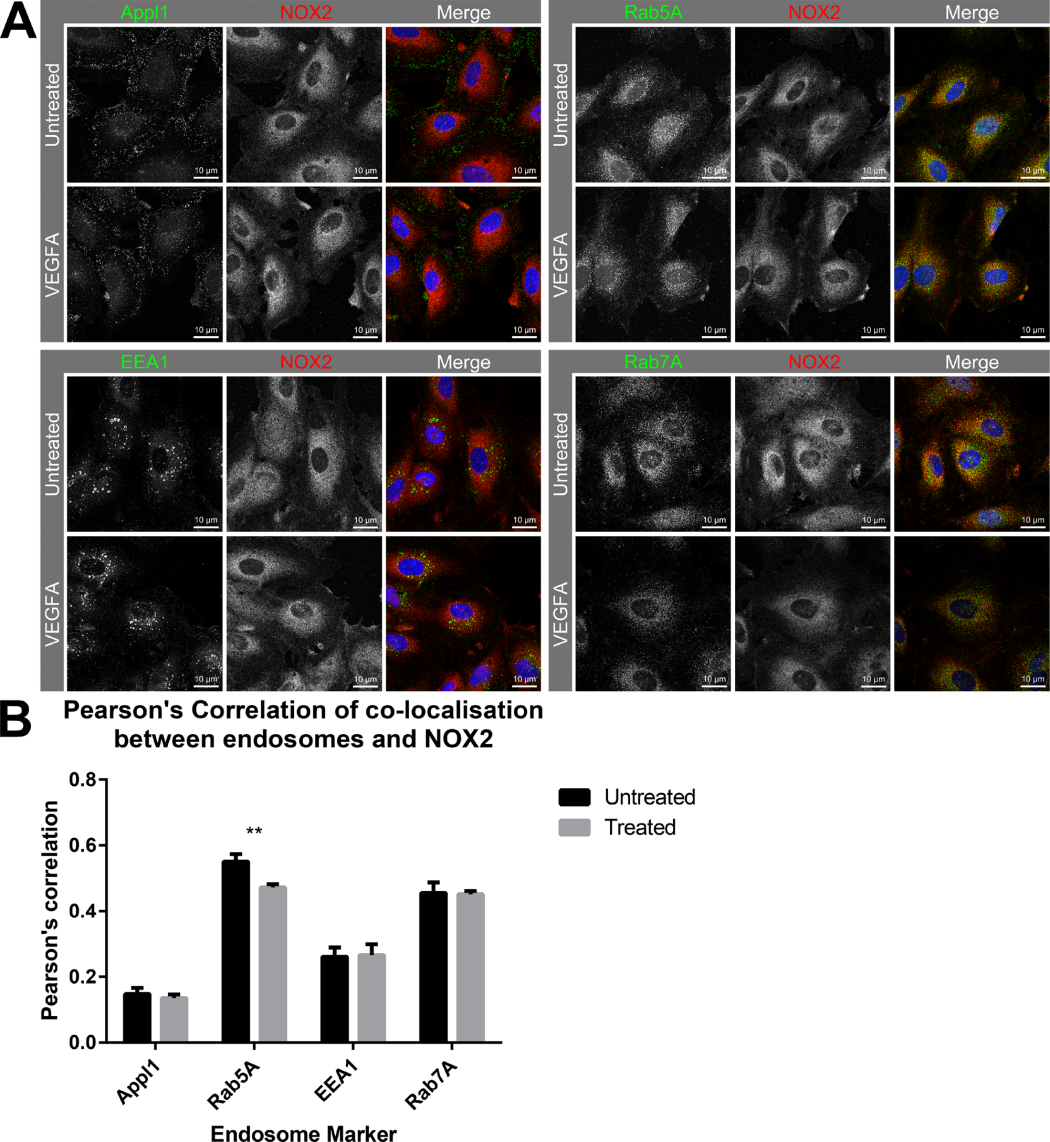
Co-localisation of NOX2 with Rab5-positive endosomes reduces in the presence of VEGF (**A**) Confocal immunofluorescent images showing NOX2 (red) with the endosome markers Appl1, Rab5A, EEA1 and Rab7A (green) co-location within HMEC-1 cells after 30 min incubation with either PBS or VEGF-A (30 ng/mL). (**B**) Graph shows the degree of co-localisation (Pearson’s correlation) within the cells. Data are mean ± SEM. ^**^P < 0.01 for Mann–Whitney unpaired *t* test.

Investigation of NOX2 localisation with endosomes in HMEC-1 with either PBS or VEGF-A treatment (30 ng/mL; 30 min) resulted in observations of minimal co-location of NOX2 with Appl1, and no effect on co-localisation resulting from VEGF-A treatment (Figure 5). Appl1 displayed distinct peri-cellular localisation compared to the perinuclear location of NOX2. Thus, we observed co-location of Rab5A with NOX2, which was significantly reduced upon treatment with VEGF-A (*P* ≤ 0.01; Figure 5). There was limited co-location of EEA1 with NOX2 and this was unchanged between untreated and treated cells. Rab7A exhibited a high-degree of co-location with NOX2, similar to that observed with Rab5A, however the degree of co-location was unaffected by VEGF-A treatment. These observations indicate that upon VEGFA stimulation, Rab5A is recruited away from NOX2-containing endosomes, likely due to the increased endocytic events.

### VEGF-A stimulates endosomal ROS in endothelial cells

**Figure 6:**
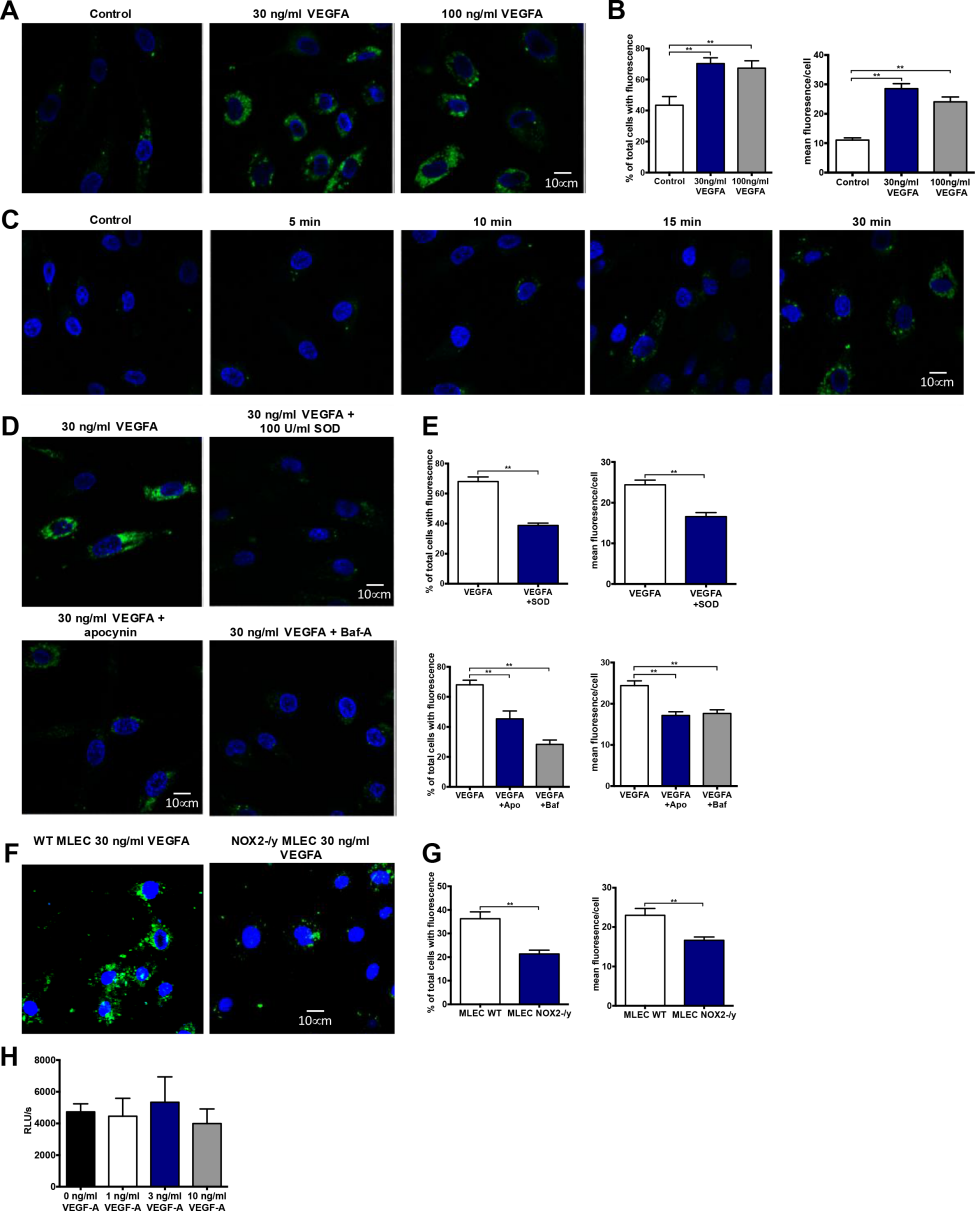
VEGF stimulates endosomal superoxide production in endothelial cells *via* a NOX2 oxidase-dependent mechanism (**A**) Confocal fluorescence images of HMEC-1 incubated with OxyBURST green for 5 min before incubating for 30 min with either PBS, VEGF-A (30 ng/mL) or VEGF-A (100 ng/mL). (**B**) Graphs showing the area of fluorescence and mean fluorescence per cell detected in each group (*n* = 5). (**C**) Confocal images of time course of endosomal ROS generation in HMEC-1 cells. Cells were incubated with OxyBURST green for 5 min and then either PBS for 30 min (control) or VEGF-A (30 ng/mL) for between 5 and 30 min (*n* = 3). (**D**) Confocal images of endosomal ROS generation after 30 min incubation with VEGF-A (30 ng/mL) and 30 min of incubation with either SOD (100 U/mL), apocynin (300 µM) or bafilomycin A (10 nM) followed by 30 min incubation with VEGF-A (30 ng/mL). (**E**) Graphs show total number of ROS-producing cells expressed as percentages of the total number of cells per group plus the mean fluorescence per ROS-producing cell (*n* = 3-5). (**F**) Confocal images of endosomal ROS generation after 30 min incubation with VEGF-A (30 ng/mL) and (**G**) their corresponding graphs showing total number of ROS-producing cells expressed as percentages of the total number of cells per group plus the mean fluorescence per ROS-producing cell for each group in wild-type and NOX2 knockout (NOX2^-/y^) mouse lung endothelial cells (MLEC cells; *n* = 4). (**H**) Extracellular H_2_O_2_ production as assessed by Amplex Red fluorescence in the absence or presence of VEGF-A (*n* = 5). Data are mean ± SEM. ^*^P < 0.05, ^**^P < 0.01 for Students unpaired *t* test (E and G) or one-way ANOVA (B and E).

Given that NOX2^-/y^ mice displayed reduced CD31+ and VEGFR2+ staining within tumours compared to WT controls we focussed on ROS production in endothelial cells. To investigate VEGF stimulated endosomal ROS generation within endothelial cells we utilised OxyBURST fluorescence microscopy. VEGF-A (30 and 100 ng/mL) generated more ROS than PBS controls (Figure 6A), with a higher mean area of fluorescence (quantified as the number of pixels of fluorescence per cell) and mean fluorescence per cell in both VEGF-A treatment groups (*P <* 0.001; Figure 6B). ROS generation within these cells was observed as punctate vesicular fluorescence within 15 min of VEGF-A addition (Figure 6C), which was consistent with an endosomal location. Addition of excess SOD (100 U/mL) almost abolished (*P <* 0.001) VEGF-dependent ROS production, both in terms of the percentage of cells generating ROS and the mean fluorescence per cell (Figure 6D and 6E). This suggested that the ROS generated within these cells was likely to be derivatives of superoxide. Furthermore, apocynin (300 µM) treatment almost abolished (*P <* 0.001) endosomal ROS production in response to VEGF-A (Figure 6D and 6E). To clarify the role of NOX2 oxidase in endosomal ROS production we utilised endothelial cells from WT and NOX2^-/y^ mice. VEGF-A (30 ng/mL) caused a significantly smaller endosomal ROS response in NOX2^-/y^ cells when compared to WT controls (Figure 6F and 6G). Treatment with bafilomycin A (10 nM) blunted ROS production in response to VEGF-A (*P <* 0.001; Figure 6D and 6E), suggesting that ROS production was dependent upon endosomal acidification and that the activation of NOX2 oxidase followed this acidification. In additional experiments, we demonstrated that VEGF-A failed to increase extracellular ROS production, as assessed by L-O12 enhanced chemiluminescence and Amplex Red fluorescence (Figure 6H). Mitochondria are significant sources of ROS in cells, however, these data provide evidence that, at least in endothelial cells activated by VEGF-A, the primary source of endosomal ROS is NOX2 oxidase. This is also consistent with the endosome ROS response to virus infection which is predominantly NOX2 oxidase-dependent [30].

### VEGF increased endothelial cell proliferation that was dependent on endocytosis, endosomal acidification and NOX2-derived H_2_O_2_

**Figure 7:**
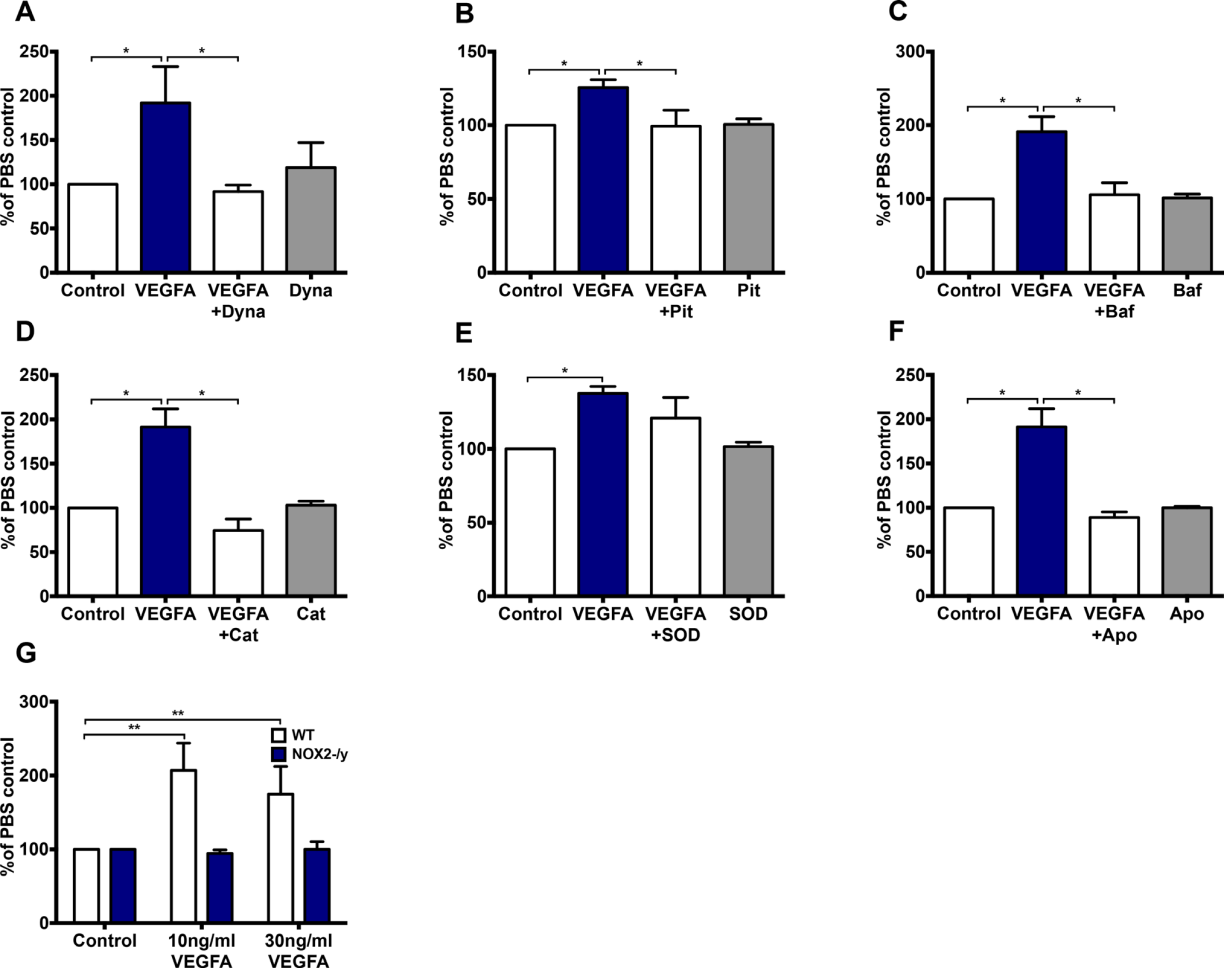
VEGF significantly increases endothelial cell proliferation in a H_2_O_2_- dependent manner and is further dependent on endocytosis, endosomal acidification and NOX2 activity (**A**–**F**) The proportion of HMEC-1 per well after 24 hr treatment with either PBS or VEGF-A (30 ng/mL) in the absence or presence of either (A) Dynasore (Dyna; 100 µM), (B) pitstop 2 (Pit; 30 µM), (C) bafilomycin A (Baf; 10 nM), (D) catalase (Cat; 1000 U/mL), (E) SOD (100 U/mL) or (F) apocynin (Apo; 300 µM). (**G**) Graph shows the effects of VEGF-A (10 and 30 ng/mL) on WT and NOX2^-/-^ mouse lung endothelial cell proliferation after 24 hr expressed as percentages of the PBS control. Data are mean ± SEM for *n* = 5-7 experiments. ^*^P < 0.05, ^**^P < 0.01, for one-way ANOVA.

In a 24-hour proliferation assay, VEGF-A (30 ng/mL) increased endothelial cell proliferation (*P <* 0.05) compared to cells grown in the presence of PBS (Figure 7A). This VEGF-dependent increase in proliferation was inhibited by dynasore (100 µM; *P <* 0.05; Figure 7A), Pitstop 2 (30 µM; *P <* 0.05, Figure 7B), bafilomycin A (10 nM; *P <* 0.05; Figure 7C), catalase (1000 U/ml; *P <* 0.05; Figure 7D) and apocynin (300 µM; *P <* 0.05; Figure 7F), indicating that VEGF-driven proliferation was reliant on endocytosis, endosomal acidification and H_2_O_2_ production. SOD failed to influence VEGF-mediated proliferation (Figure 7E). Neither dynasore, pitstop 2, bafilomycin A, catalase, apocynin nor SOD alone had any effect on cell proliferation (Figure 7A–7F). Both 10 and 30 ng/mL VEGF-A significantly increased (*P <* 0.01) cell proliferation after 24 h in WT controls but not in NOX2^-/y^ cells (Figure 7G).

### VEGF-mediated endothelial ROS production and proliferation were independent of NOX1

**Figure 8:**
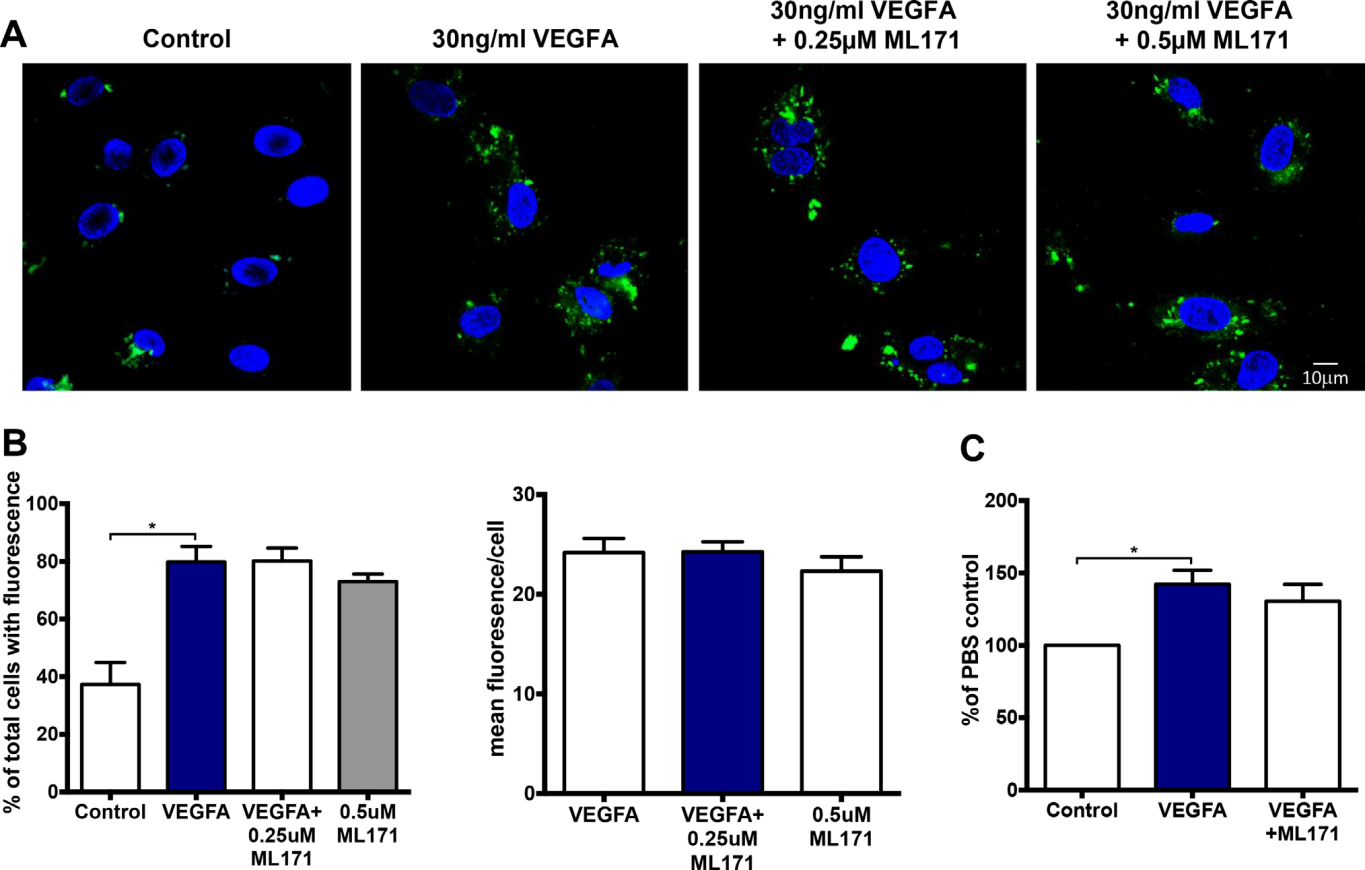
VEGF-dependent endosomal ROS production and proliferation occurs independently of NOX1 (**A**) Confocal immunofluorescent images of endosomal ROS production within HMEC-1 in the presence of either PBS, VEGF-A alone or VEGF-A (30 ng/mL) with ML171 (either 0.25 or 0.5 µM). (**B**) Graphs show total number of ROS-producing cells expressed as percentages of the total number of cells plus the mean fluorescence per ROS-producing cell for each group (*n* = 6). (**C**) The proportion of HMEC-1 per well after 24 hr treatment with either PBS alone, VEGF-A (30 ng/mL) alone or VEGF-A (30 ng/mL) plus ML171 (0.5 µM). Data are expressed as a percentage of the PBS control. Data are mean ± SEM for *n* = 3–6 experiments.

To investigate the effect of NOX1 inhibition on endothelial proliferation and ROS production, we performed OxyBURST and proliferation assays in the presence of the NOX1 inhibitor, ML171. Neither 0.25 µM nor 0.5 µM ML171 had any inhibitory effect on the number of ROS-producing cells or the mean area of fluorescence produced by 30 ng/mL of VEGF-A (Figure 8A and 8B). Furthermore, ML171 (0.5 µM) did not affect VEGF-A-mediated endothelial cell proliferation after 24 h (Figure 8C).

## DISCUSSION

NOX2 oxidase was expressed at higher levels in human primary prostate cancers when compared to normal tissue, suggesting that this ROS generating enzyme plays a role in tumour development. Indeed, using a mouse model of prostate cancer, we show evidence that NOX2 oxidase is crucial for angiogenesis and tumour development. Furthermore, we provide evidence that NOX2 oxidase is expressed in the endosomal compartments of endothelial cells and non-malignant prostate and at higher levels in malignant prostate cancer cells; providing a culprit source of ROS that transduces VEGF-dependent signalling. Given that other growth factors like basic fibroblast growth factor (FGF2) and hepatocyte growth factor (HGF) also activate proliferation following internalisation of their receptors into endosomes [11], our data suggests that endosomal ROS may influence growth factor receptor signalling *per se*, representing a critical point of convergence of this type of signalling, which is then able to promote tumour development.

The NADPH oxidase family of enzymes contributes to many forms of angiogenesis, such as in re-vascularization of the brain after stroke [19] and in developing a complete vasculature in the growing foetus [13]. However, the role of NADPH oxidases in the setting of cancer angiogenesis has remained unclear. The RM1 murine prostate cancer cell line was originally developed by overexpressing oncogenes in murine urogenital sinus cells before implanting them under the kidney capsule to yield prostate carcinomas [29]. This cell line allowed us to determine the role of NOX2 oxidase in the tumour angiogenesis *in vivo*. Here, we show for the first time that genetic deletion of NOX2 resulted in reduced tumour growth in the prostate following orthotopic administration of the RM1 prostate cancer cell line. In some cases, there was a complete failure of tumour growth in NOX2^-/y^ mice, indicating that ROS production has a fundamental role in tumour development. CD31^+^ cells are a marker of angiogenesis, and their reduced number in NOX2^-/y^ tumours implied that the lack of tumour development might be attributed to less angiogenesis. In support of reduced angiogenesis in the NOX2^-/y^ mice was the reduced expression of the VEGFR2 within the tumours. Pharmacological treatment with the NOX2 oxidase inhibitor and H_2_O_2_ scavenger apocynin also caused a substantial reduction in tumour development. The effect of apocynin was most likely on the tumour microenvironment rather than on the RM1 cells, as apocynin failed to influence RM1 cell proliferation *in vitro*.

ROS can promote endothelial cell proliferation in response to growth factors such as VEGF-A, but the subcellular location of ROS production has not been defined. To establish a subcellular role for ROS in tumour growth it is imperative to have an understanding of the growth factor receptor signalling mechanisms that underpin cell proliferation. Tumour cells release growth factors that stimulate endothelial cell proliferation, which is a key process in tumour angiogenesis [22]. We therefore examined the signalling arising from VEGF-A interacting with its cognate receptor VEGFR2 in endothelial cells. Ligand stimulation of the VEGR2 results in rapid internalisation of the receptor complex from the plasma membrane into early endosomes, triggering ERK1/2 and AKT phosphorylation to drive cell proliferation [11]. Following exposure to VEGF-A, the endothelial cell VEGFR2 co-located with EEA1-positive early endosomes and within 5 minutes resulted in MEK and ERK1/2 phosphorylation [11]. The VEGFR2 and EEA1 co-location in response to VEGF-A was almost abolished by dynasore and Pitstop 2, which suppressed MEK and ERK1/2 phosphorylation as well as cell proliferation [11]. In the present study, we confirmed in endothelial cells that VEGF-A exposure initiates VEGFR2 trafficking through the endocytic network, as it co-located with EEA1 within minutes of exposure to the agonist. There was also a large proportion of VEGFR2 that did not co-locate with EEA1, suggestive of VEGFR2 compartmentalization in other subsets of endosomes. We also showed that NOX2 co-located with endosomal markers including Rab5A and Rab7A in endothelial cells, placing this isoform of NADPH oxidase within early and late endosome compartments, similar to the reported locality of the VEGFR2. The reduction of NOX2 and Rab5A co-location upon treatment is likely due to recruitment of Rab5A to newly initiated endocytic events from VEGFA uptake, but we could not exclude the possibility that this reflects a depletion of Rab5 positive endosomes (e.g. as part of an endosome maturation process). Additionally, we showed that endosomal ROS production occurred after 15 minutes of VEGF-A exposure and was dependent on endosomal acidification. These findings indicated that ROS production was occurring within endosomes, as this process only occurs after internalisation of the receptor into the endosome and was inhibited if endosomal acidification was abrogated. However, we could not rule out delivery to other specialist endosomal compartments or indeed amphisomes and autophagic compartments as each of these vesicles also involve interactions with endosomal machinery and compartments. Interestingly, there was no differential NOX2 association with the endosomes (in most cases, if not reduced) after VEGF treatment. Whilst there is co-location of NOX2 with endosomal markers, the enzyme still needs to be activated by a complex process to generate ROS. For example, VEGF-A binding to the VEGFR2 receptor will drive a Rac-1-dependent process that ultimately aids in the assembly and activation of Nox2 oxidase [6]. Nox2 oxidase is a multi-subunit enzyme complex that requires a series of activation steps to promote full enzyme assembly and ROS generation. Importantly, apocynin and the deletion of endothelial NOX2 both inhibited endosomal ROS production. Finally, VEGF-A failed to influence extracellular ROS levels as measured by Amplex Red fluorescence and L-O12 enhanced chemiluminescence, which was consistent with the endosome being the primary site of ROS generation in response to VEGF-A.

Having demonstrated that VEGF-A increases endosomal ROS in endothelial cells, our next step was to examine if this ROS contributes to cell proliferation. Endosomal ROS was sensitive to bafilomycin A, which also suppressed proliferation, suggesting that endosomal ROS has a direct impact on cell proliferation. Catalase, which is a large enzyme that is internalized by endocytosis, also suppressed cell proliferation supporting the concept that the endosome was the site of ROS production impacting on cell proliferation. However, the observation that catalase, but not SOD, suppressed cellular proliferation led us to speculate that H_2_O_2_ and not superoxide anion *per se* promoted the cell proliferation (N.B. to prove this is difficult as methods to detect localised H_2_O_2_ or superoxide are not currently possible).

We show evidence that VEGFR2 and NOX2 are expressed in early and late endosomes in endothelial cells that generate ROS to modulate cell proliferation, which is likely to play an important role in the processes of tumour angiogenesis. We also examined the subcellular location of NOX2 in human non-malignant and malignant prostate cancer cells. Similar to endothelial cells, NOX2 expression strongly co-located with Rab5A and Rab7A, signifying an early and late endosome location. In addition, NOX2 was co-expressed in Appl1 and EEA1 positive endosomes. Strikingly, there was a significantly higher degree of co-location of NOX2 with all the endosome markers tested in the metastatic prostate epithelial cells, LNCaP versus PNT1a non-malignant cells. The altered endosome biology in prostate cancer cells, and in particular the increased NOX2 expression in endosomes, is likely to contribute to elevated ROS generation in prostate cancer.

Our study provides evidence that NOX2 oxidase promotes tumour angiogenesis, however, we are not ruling out a contribution from other NOX1 isoforms including the NOX1 and NOX4 oxidases. NOX1 oxidase expression and activity is increased in mouse primary and human endothelial cells upon VEGF and FGF exposure, and NOX1 silencing decreases endothelial cell migration and tube formation through the inhibition of PPARα, a regulator of NF-κB [10]. Overexpression of NOX1 in fibroblasts and in carcinoma cells induces an angiogenic switch mediated by increased production of VEGF and MMPs [1]. VEGF mRNA was upregulated by NOX1 in tumours, and both VEGFR1 and VEGFR2 were highly induced in vascular cells of NOX1-expressing tumours [1]. However, our findings show that both the increases in endothelial cell proliferation and endosomal ROS generation in response to VEGF-A were unaffected by the selective NOX1 inhibitor, ML171. Thus, the NOX1 tumour-promoting effects were likely to be due to activation of pathways distinct from NOX2. Indeed, as opposed to NOX2, silencing and inhibition of NOX1 activity failed to affect cell proliferation [10] but instead resulted in reduced cell migration [10]. NOX4, the only constitutively active NOX isoform, is involved in a myriad of endothelial cell processes, including proliferation and angiogenesis (for review see [3]. NOX4 promotes tube formation and cell proliferation in response to TGF-b1, as well as TGF-b1-stimulated angiogenesis *in vivo* [4]. We previously showed that both downregulation of NOX4 and the addition of the H_2_O_2_ scavenger, catalase, in endothelial cells decreased ERK1/2 phosphorylation, ROS production and cell proliferation, but had no effect on cell death [25]. The dose and duration of H_2_O_2_ signalling need to be defined in the future, as this may specifically alter the endothelial proliferation, senescence and apoptosis phenotypes. In the present study we have revealed from the human microarray databases that NOX4 expression is elevated in prostate cancer compared to normal tissue, however, from the Tomlins review it appears that NOX4 is elevated predominantly in metastatic prostate cancer and not in primary prostate cancer. Overall, future studies need to delineate specific roles of individual NOX isoforms *in vivo* with an emphasis on subcellular localization and compartmentalized redox signalling mechanisms in the context of both indolent/benign tumours and aggressive metastatic cancers, not only restricted to prostate cancer but other cancers such as lung, breast, ovarian and skin.

To summarise, we have evaluated the role of NOX2 oxidase in angiogenesis and tumourigenesis in a mouse model of prostate cancer. Deletion of NOX2 resulted in a profound reduction in tumour development and angiogenesis. Moreover, NOX2 co-located with several markers of early and late endosomes to promote endosomal ROS generation and proliferation in response to VEGF-A. Given that NOX2 is expressed highly in human prostate cancers, this study provides novel *in vivo* evidence for a molecular mechanism activated by the cancer-promoting VEGF pathway, and hence a rationale for therapeutic inhibition of endosomal NOX2 oxidase in prostate cancer.

## MATERIALS AND METHODS

### Patient cohorts

The Tomlins [31] cohort was chosen for its curation of multiple disease stages of prostate cancer. Analysis of tissue samples from this cohort was previously performed using the Chinnaiyan Human 20K Hs6 array [31] and was retrieved from NCBI GEO (accession number GSE6099). It is comprised of 18 non-malignant tissues, 13 prostatic intraepithelial neoplasia’s (PIN), 30 primary prostate cancers and these were obtained from radical prostatectomies and 19 metastatic cancer tissue samples were obtained from hormone refractory metastases in the liver, lung or lymph tissue [31]. The Grasso cohort 12 was comprised cancer (*n =* 59) and normal tissue (*n =* 28) that were obtained from treatment naïve men at the time of prostatectomy [12]. Analysis of tissue samples from this cohort was previously performed using Agilent Whole Human 44k element arrays and was retrieved from NCBI GEO (accession number GSE35988).

### General cell culture

RM1 cells, a murine prostate carcinoma androgen-insensitive cell line, were derived by transformation from the genital ridge of C57BL6/J mice. Cells were grown in 75 cm^2^ flasks in Dulbecco’s Modified Eagle Medium (DMEM; Invitrogen, New York, NY, USA) with glucose (1000 mg/L) and 10% Foetal Bovine Serum (FBS; Sigma, Australia) and incubated with 5% CO_2_ at 37° C. Wild-type and NOX2^-/-^ mouse immortalized lung endothelial cells (MLEC) were obtained from Dr. Hitesh Peshavariya of Melbourne University. Both MLEC and human microvascular endothelial cells (HMEC-1) were grown in 25 cm^2^ flasks in complete Endothelial Growth Basal Medium 2 (EBM-2; Lonza) and incubated with 5% CO_2_ at 37° C. Cells were passaged when they reached confluency using trypsin (0.15%; Invitrogen, New York, NY, USA). Cell viability was determined using the Trypan Blue (Sigma, Australia) cell exclusion test.

### Endothelial cell proliferation assay

1 × 10^5^ HMEC-1/ MLEC were plated in duplicate in 6-well plates for 24 hr at 37° C, 5% CO_2_ in EBM-2 media containing 5% FBS and ascorbic acid only. Cells were washed with PBS and the media was replenished before VEGF-A (concentrations of 1, 3, 10 and 30 ng/mL for initial proliferation characterisation and 30 ng/ml thereafter for MLEC and inhibitor studies) or PBS was added to each well for 24 hr and proliferation was assessed using the Trypan Blue exclusion test. For the inhibitor studies, duplicate wells were seeded with 1 × 10^5^ HMEC-1 cells, as above, and the respective inhibitors catalase (1000 U/ml; Sigma), dynasore (100 µM; Sigma), bafilomycin (10 nM, Sigma), SOD (100 U/ml, Sigma), Pitstop 2 (30 µM), apocynin (300 µM, Sigma) or the NOX1 inhibitor ML171 (0.5 µM) were added 30 mins before adding VEGF-A. Each inhibitor group had its own control consisting of the same procedure but adding PBS in place of VEGF-A for 24 hr. Proliferation was again assessed using the Trypan Blue exclusion test.

### Endosomal ROS production

5 × 10^4^ HMEC-1 were seeded onto coverslips in 24 well plates for 24 hr at 37° C, 5% CO_2_ in EBM-2 media containing 5% FBS and ascorbic acid only. Cells were washed with PBS and the media was replenished before either PBS or one of the inhibitors used in the endothelial proliferation assay and 50 µM OxyBURST Green reagent (Life Technologies, Australia, Catalog number: D2935) was added 5 mins before a 30 min incubation at 37° C, 5% CO_2_ with either PBS or VEGF-A (30 or 100 ng/mL). Cells were washed with PBS and left to sit in 4% paraformaldehyde for 15 min before receiving 3 × 10 min washes in 0.01 M PBS. Coverslips were removed from wells and mounted with DAPI (Sigma, Australia) onto baked slides. Fluorescence was detected using a Nikon C1 confocal microscope and results analysed using Image J software (version 1.45, National Institutes of Health).

### Amplex red

HMEC-1 were collected by trypsinization and seeded in 96-well plates at a density of 5 × 10^5^ cells. VEGF-A (0, 1, 3 or 10 ng/mL) was added to the cells for 10 min, and then the Amplex Red reaction mixture was added to give final concentrations of 0.005 U/mL horseradish peroxidase and 25 µM Amplex Red. Fluorescence was recorded for 60 min at 37° C with excitation and emission wavelengths of 550 nm and 600 nm respectively. The amount of ROS generated was calculated from a hydrogen peroxide standard curve ranging from 5 µM to 0.156 µM, which was included on each plate. The generation of hydrogen peroxide by the xanthine/xanthine oxidase was performed in PBS supplemented with xanthine oxidase (0.004 U), ethylenediaminetetraacetic acid (EDTA) (0.3 mM), HRP (0.005 U/mL) and Amplex Red 0.025 mM. The reaction was started by the addition of xanthine (0.5 mM).

### Triple labelling immunofluorescence

5 × 10^4^ HMEC-1 were seeded and after 24 hr in media, cells were washed with PBS and the media was replenished before a 30-min incubation at 37° C, 5% CO_2_ with PBS or VEGF-A (30 ng/mL). Cells were then washed with PBS and left to sit in 4% paraformaldehyde for 15 mins before receiving 3 × 10 min washes in 0.01 M PBS. Cells were then incubated in antibody diluent (0.25% Triton X; Sigma, Australia) for 10 mins before receiving 3 × 5 min washes in 0.01 M PBS followed by 2 hr incubation in 10% goat serum (Sigma, Australia) at room temperature. After further 3 × 10 min washes in 0.01 M PBS, cells were incubated in primary antibodies including anti-EEA1 antibody (1:1000; Sapphire Biosciences Pty. Ltd, Australia), rabbit mAb VEGF receptor 2 antibody (1:1000; Cell Signaling, Australia) and mouse anti-gp91phox antibody (1:1000; BD Biosciences, Australia) overnight at 4° C. Cells were then given 4 × 10 min washes in 0.01 M PBS before being incubated with secondary antibodies (1:1000 for each of Alexa fluor 647 goat anti-rabbit and 488 goat anti-mouse, both Life Technologies) for 2 hr at room temperature. After a final series of 4 × 10 min washes cells were mounted with DAPI on baked slides. Fluorescence was detected using a Nikon C1 confocal microscope and results analysed using Image J software (version 1.45, National Institutes of Health). All immunohistochemistry was assessed by two observers blinded as to the treatment groups throughout the analysis process and all of the appropriate controls were performed, in that all combinations of primary and secondary antibodies were used to ensure no cross reactivity occurred (To *et al.*, 2017).

### Syngeneic model of orthotopic mouse prostate tumour

Male NOX2^-/y^ and C57BL6/J mice were obtained from Monash Animal Services (Clayton, Victoria, Australia). All mice were 8–12 weeks of age and normal chow and drinking water were provided ad libitum. The Monash University Animal Research Platform and Animal Ethics Committee approved the study.

Mice were anaesthetized via inhalation of a 5% isoflurane/95% oxygen air mixture, and an incision made through the skin and muscle of the abdomen to expose the bladder and, underneath, the prostate. A cell suspension (10 µL) containing 5 × 10^3^ RM1 cells in DMEM media plus 10% FBS was injected into the prostate and the wound closed. Sham mice received 10 µl of DMEM media plus 10% FBS only. Tumours were allowed to develop over time periods consisting of 3, 7, 10 and 14 days. At each time point, mice were sacrificed via CO_2_ asphyxiation. Blood was collected via cardiac puncture and the prostates were removed for gross morphological analysis and placed in 10% formalin for CD31+ immunohistochemistry. Tumour weights were recorded as the prostate plus its associated tumour (or prostate only in the case of sham mice) and seminal vesicles.

### *In vivo* pharmacological inhibitor studies

Prostate tumours were established as per the above protocol. To address VEGFR2, mice were randomly assigned to two treatment groups: vehicle treated (10% DMSO in 100 µl saline injection) or VEGFR2 inhibitor Ki8751 treated (20 mg/kg dissolved in 10% DMSO in 100 µl saline injection; Selleckchem) and were administered their respective treatment intraperitoneally from days 10 to 14. At day 14, mice were culled via CO_2_ inhalation and the prostates were removed for gross morphological analysis. For apocynin studies, mice in each study were randomly assigned to two treatment groups: vehicle treated (10% DMSO in 100 μL saline injection plus 0.1% DMSO in drinking water) or apocynin treated (50 mg/kg dissolved in 10% DMSO in 100 μl saline injection plus 500 mg/L dissolved in 0.1% DMSO of drinking water) [27]. Mice were treated from days 10 to 14, and each drinking water solution was made up fresh daily. At day 14, mice were culled via CO2 inhalation and the prostates were removed for gross morphological assessment, CD31+ immunohistochemistry and flow cytometric analysis.

### CD31+ immunohistochemistry

After fixing in 10% formalin, prostate samples were embedded in paraffin and cut at 4 µm thickness. The sections were then incubated with primary antibody (rabbit anti-mouse CD31; 1:50; Abcam Co.) overnight at 4° C followed by a 2 h staining with a peroxidase-labeled polymer conjugated to goat anti-rabbit immunoglobins (Dako Australia Pty, Ltd). The reaction was developed with 3,3’-diaminobenzidine (DAB; Dako Australia Pty, Ltd) and finally counterstained with hematoxylin [24]. Quantification of CD31^+^ staining was performed using light microscopy. Sections were analysed on digitised colour images and evaluated by counting the number of positive staining vessels in five fields within the tumour, chosen at random. Vessels were defined as any brown-staining (DAB immunoperoxidase stain with anti-CD31 antigen) cell cluster clearly separated from any adjacent vessels [7].

### Statistical analysis

Statistical analysis was carried out using Students *t*-test or one-way ANOVA. All values of *P <* 0.05 were considered to indicate statistical significance. Results are expressed as mean ± standard error of the mean (SEM).

## SUPPLEMENTARY MATERIALS FIGURE


