# Supplementary material for: NOX2 oxidase expressed in endosomes promotes cell proliferation and prostate tumour development
Source: Oncotarget. 2018 Oct 23;9(83):35378–93. doi: 10.18632/oncotarget.26237 (PMC6226044; doi:10.18632/oncotarget.26237)
Supplement: Supplementary file 1 [file oncotarget-09-35378-s001.pdf]

NOX2 oxidase expressed in endosomes promotes cell proliferation and prostate tumour development

SUPPLEMENTARY MATERIALS

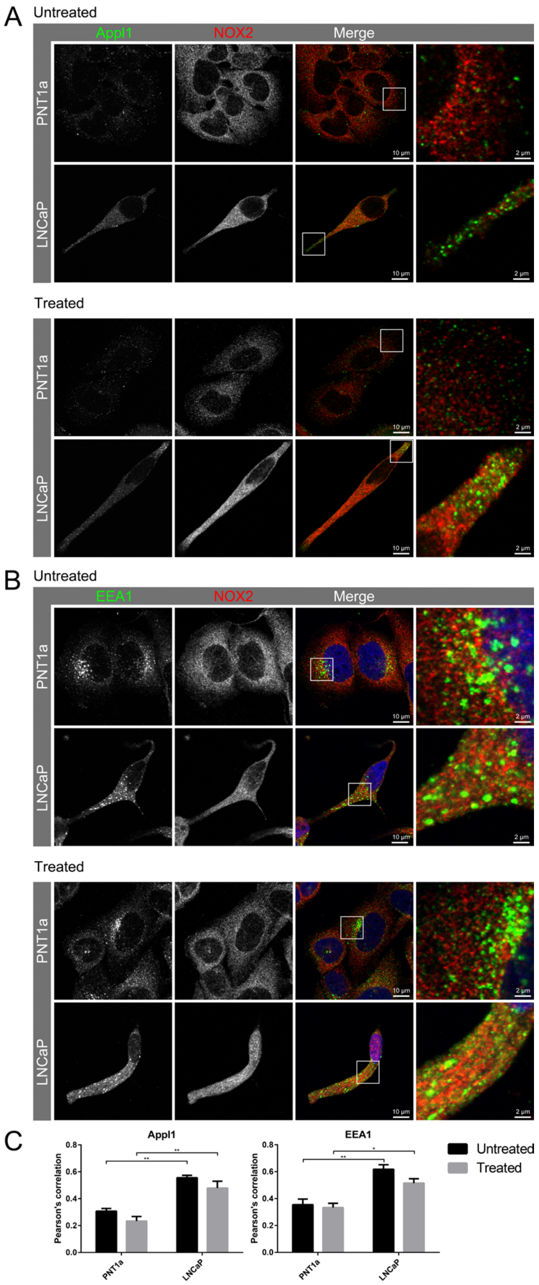

Supplementary Figure 1: Co-location of NOX2 with endosome markers in response to VEGF-A treatment. (**A**) Confocal fluorescent images showing co-located NOX2 (red) with endosome markers (green) Appl1 and EEA1 in non-malignant (PNT1A) and malignant (LNCaP) human prostate cancer cells and in LNCaP post-VEGF-A treatment. (**B**) Graphs show the degree of co-location of NOX2 with the endosome markers Appl1 and EEA1 in VEGF-A-treated and untreated PNT1a and LNCaP cells. Data is representative of 6 randomly selected cells and are shown as mean ±SEM. $^{*}P < 0.05$, $^{**}P < 0.01$ for two-way ANOVA.
